# The Role of Motor Imagery in Predicting Motor Skills in Young Male Soccer Players

**DOI:** 10.3390/ijerph18126316

**Published:** 2021-06-10

**Authors:** Dariusz Zapała, Emilia Zabielska-Mendyk, Andrzej Cudo, Marta Jaśkiewicz, Marcin Kwiatkowski, Agnieszka Kwiatkowska

**Affiliations:** 1 Department of Experimental Psychology, The John Paul II Catholic University of Lublin, 20-950 Lublin, Poland; zabielska.emilia@op.pl (E.Z.-M.); andrew.cudo@gmail.com (A.C.); martajaskiewicz007@gmail.com (M.J.); 2 Sport and Minds, 21-030 Motycz, Poland; m.kwiatkowski@sportandminds.pl (M.K.); a.kwiatkowska@sportandminds.pl (A.K.)

**Keywords:** motor imagery, young athletes, soccer, reaction time, coordination

## Abstract

The study aimed to find out whether the imagery ability within the two subcomponents of motor imagery (visual and kinesthetic) allows predicting the results in simple response time task and eye–hand coordination task in a group of young male soccer players (9–15 years old). Non-specific simple response time and eye–hand coordination play a key role in predicting specific sports performance level. Participants performed Reaction Time Task, Eye–Hand Coordination Task, and completed Motor Imagery Questionnaire–Revised. Data were submitted to the structural equations analysis based on the maximum likelihood method in order to estimate a structural model of relationship between variables. Results indicate visual rather than kinesthetic motor imagery is associated with non-specific motor skills. Higher scores on the visual motor imagery scale were observed to correlate with faster reaction times and better coordination in the study group. This supports the idea that during learning a new perceptual-motor-task the visual control is required. Results provide the evidence for the specific role of the third-person perspective imagery in young athletes playing soccer.

## 1. Introduction

Motor imagery (MI) is defined as the mental simulation of a movement without any real action [1]. MI, also called “visualization” or “mental practice”, is the common form of mental rehearsal used in athletics, practiced in a very diverse and unsystematic way [2,3,4,5]. Studies investigating groups of elite, world-class athletes have found that rates of imagery use range from 70% to 99% and that MI is used by approximately 94% of sports coaches [6,7]. The frequency of motor imagery use increases with competitive level [2], and differentiates amateurs from experts [8] and successful from unsuccessful players [9]. Moreover, motor imagery training can affect motor behavior, like muscle strength [10,11] or graphomotor trajectorial learning [12].

MI may be subdivided into visual motor imagery (VMI) and kinesthetic motor imagery (KMI). If there is no specific instruction given, the subject may either imagine seeing himself, or another person, performing an action (exterior view from the third-person perspective) or a self-performed action which is accompanied by a feeling of actually performing the task (kinesthetic experience from the first-person perspective) [13]. Some studies have shown that these two types of imagery have different effects on performance in sport. Visual motor imagery is more effective if the participant has to focus precisely on the target, like in darts [14] or beach volleyball [15]. On the other hand, kinesthetic imagery is beneficial when it is necessary to correct motion parameters based on sensory feedback, for example, in tennis [16]. In practice, people often combine KMI and VMI and the interoceptive and proprioceptive sensations involved in first-person perspective are modulated by visual sensations (for review: [7]). Both KMI and VMI share similar but not entirely the same neural networks (for review: [17,18,19]).

Most research on visualization in sports concerns adult athletes. Only a limited number of studies have examined motor imagery in children. Some studies using the hand laterality judgment paradigm indicate that children of 5 to 7 years old can already use motor imagery in an implicit motor imagery task [20,21]. Molina, Tijus and Jouen [22] show that the motor imagery process can be seen about 7 years old when children are ready to think about themselves in action. However, Smits-Engelsman and Wilson [23], using the mental chronometry paradigm, reported that the motor imagery abilities do not emerge before 10–12 years of age. According to the results of the abovementioned studies on motor imagery in children, the exact age of its emergence is not clear (for review: [24]).

Although the age in which the motor imagery emerges is difficult to define, the research shows that the level of performance in the motor imagery tasks is higher in older than younger children. In this context, the age differences were clearly shown in the study by Butson [25] where less than 40% of 5–6-year-old children completed the hand rotation task, compared to 80% of 7–8-year-old children, and 90% of 9 years old and older. Accuracy and efficiency improved with age—11-year-old participants were significantly more accurate than 7- and 8-year-olds. Caeyenberghs et al. [26] compared 7- and 8-year-olds, 9- and 10-year-olds, and 11- and 12-year-olds in a back-view hand laterality judgment task and found that the younger children responded slower and less accurately than older children. Surprisingly, there was no interaction between the rotation angle of hand and age, suggesting that children can use similar strategies for solving the task across age. Additionally, Funk et al. [20] suggested that children are more involved in the motor imagery strategy than other strategies (non-motor imagery) to solve the task and the involvement of other strategies increases with age.

Also, many studies based on the mental chronometry paradigm indicate differences in the reaction time of movements and better accuracy in older children. Motor imagery duration was closer to actual execution in children aged 11 and 12 years old than younger children (7- and 8-year-olds) [27]. In the study by Skoura et al. [28] only the 10-year-old children showed significantly higher temporal congruence than the 8-year-olds (the groups of 6 and 8 years did not differ concerning temporal congruence between actual performance and imagined performance). Comparisons of adolescents and adults also showed that the strength of association between hand movement execution and imagery increased with age, indicating the development of action representation from adolescence to adulthood [29].

Motor imagery in children and adolescents was tested not only with the use of behavioral tests, like mental chronometry or hand laterality judgment, but also with the self-description questionnaires. Using the Sport Imagery Questionnaire–Children’s Version Gregg and Strachan [30] showed that younger athletes (6–8 years) were more likely to use motivational-specific (goal-oriented) images than older, who use imagery to the development of game strategies, defensive structure, etc. This means that older children (>10 years) use their motor imagery to plan activities directly related to sports performance, while younger children focus on the emotional and motivational side (earning points). There was also a positive relationship observed between motor imagery and self-confidence in and the self-assessment of one’s abilities in the group of 9–12-year-old children [31].

Moreover, imagery training in a group of children, just like in the case of adults, can be used to improve performance. In a study by Simonsmeier et al [32], four-week-long imagery training in a group of female gymnasts aged 7 to 15 had positive effects on performance only for the high-expertise athletes. Similar results were also observed in tennis players aged 7 to 10 years [33] and female gymnasts aged 7 to 14 years [34].

In summary, children aged 7 and above are able to perform tasks related to the use of motor imagery. However, it is not known how the motor imagery is related to the basic motor activities, like the speed of motor reaction to an event or coordination skills, which are one of the key abilities for sports practice [35,36,37,38]. Consequently, the study aimed to investigate the relationship between self-reported motor imagery skills in young athletes (7 to 15 years) and their fundamental motor skills measured by reaction time (RT) and eye–hand coordination. In addition, the technical and tactical assessment of the player by the coaches was taken into account as a subjective measure of a player’s rating. It was expected that kinesthetic motor imagery level and visual motor imagery level will be negatively related to RT.

Additionally, it was expected that kinesthetic motor imagery and visual motor imagery would positively relate to coordination. There should be a correlation between KMI and VMI observed, in as much as both constructs represent a related theoretical concept. Also, the study aimed to find out if RT and coordination are related to a trainer’s assessment of technique and tactics in young athletes.

## 2. Materials and Methods

### 2.1. Participants

In the study participated 71 preadolescent boys aged 9–15 years old (*M* = 11.68, *SD* = 1.75), who are young athletes recruited from three soccer teams. The group consisted of 18 boys aged 9–10 years, 28 boys aged 11–12 years, and 25 boys aged 13–5 years old. Their sports experience ranged from one to eight years of soccer training (*M* = 4.35, *SD* = 1.62). 64 of the subjects were right-handed, five were left-handed, and there is no information on handedness of two boys. Assessment of laterality was determined based on the results of Zazzo and Galifret Granjon’s Task to Examine Laterally [39,40]. The study was conducted in accordance with the Declaration of Helsinki. Additionally, approval of a local ethics committee was obtained for this study.

### 2.2. Measures

#### 2.2.1. Reaction Time Task

**Figure 1:**
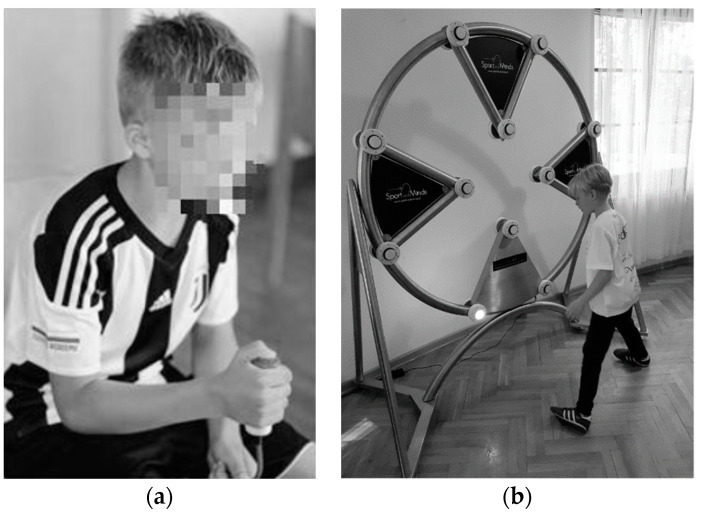
(**a**) The measurement of the simple reaction time in response to a visual cue. (**b**) Performance of the Eye–Hand Coordination Task. The subject presses the disappearing points in the shortest possible time using both hands.

In the Reaction Time Task young athletes had to press the key as fast as they had seen the appearance of a red light using the dominant hand only (see Figure 1a). There were 20 trials presented during 80 s in two runs for each participant—mean RT from each run was calculated, and shorter RT was registered. RT was measured with 1 ms accuracy. To measure the reaction time the MCZR/ATB 1.0 (ATB INFO-ELEKTRO, http://info-elektro.pl, accessed on 8 May 2021) was used.

#### 2.2.2. Eye–Hand Coordination Task

The Eye–Hand Coordination Task lasted for 60 s, and during this time, young athletes had to respond with the left or right hand as fast as they had seen the appearance of white light at one of 12 buttons distributed in the space in front of them (see Figure 1b). The result was a number of hits. In addition, a number of misses were also registered. The sequence of the lights was always the same for each participant, and each participant had two runs—the better result was registered. To measure coordination BLINK device (www.blink.pro, accessed on 8 May 2021) was used.

#### 2.2.3. Motor Imagery Questionnaire–Revised

Participants were asked to complete the polish experimental translation of Motor Imagery Questionnaire–Revised second version (MIQ-RS), which consists of two subscales with seven items for visual motor imagery scale and seven items for kinesthetic imagery scale [41].

#### 2.2.4. Short Survey

Players also completed a short survey with questions about age, handedness, and sports experience. Trainers were asked for an assessment of the tactics and technique of each young athlete. Tactics was defined as using the techniques that force the opponent to leave their own strategy and switch to one that does not ensure success. Whereas technique, as sensory motor skills that allow achieving sports goals [42]. Trainers rated both parameters at a 1–10 scale, where 1 indicated inferior performance and 10 indicated excellent performance (see Appendix A: Table A1).

### 2.3. Procedure

Participants were approached after some scheduled athletic practice time and asked to complete the MIQ-RS and do the Reaction Time Task (RT) and the Eye–Hand Coordination Task (EHCT). The questionnaire was conducted independently by each athlete, though the researcher administering the questionnaire was reading the instruction and providing clarification if needed due to the young age of the participants. After completing the MIQ-RS, subjects have done the RT and the EHCT.

## 3. Results

Analysis of structural equations based on the maximum likelihood method was applied to estimate a structural model [43]. The following statistics were applied as measures of model fit: χ^2^, χ^2^/df, RMSEA, SRMR, GFI, CFI, and NFI [42]. Statistically insignificant (*p* > 0.05) chi-square values may suggest that the proposed model fits the dataset well. The value of chi-square/df ratio lower than 2 suggests a good fit to a dataset [44]. Likewise, values of RMSEA lower than 0.05 show a good fit of the model [44]. Values of GFI, CFI, and NFI higher than 0.95 allow a conclusion that a model fits well to a dataset [45]. Also, bootstrapping method (5000 sample) with bias-corrected percentile method was used to estimate a regression weights, correlations, and direct effect with 95% confidence interval [44]. In addition, taking into account the age of the respondents, the reaction time was referred to the norms developed for the Polish population [46].

**Figure 2:**
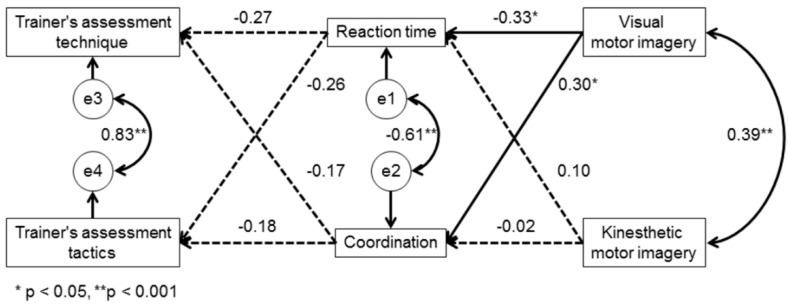
Structural model of relations between motor imagery, RT, coordination, and the trainer’s assessment.

Based on the calculations carried out, it was shown that the model fits well the data: χ^2^ (df = 4) = 1.93; *p* = 0.750; χ^2^/df = 0.48; RMSEA = 0.000, SRMR = 0.033, GFI = 0.991, CFI = 1.000, and NFI = 0.987. The visual motor imagery was negatively and significantly related to reaction time (β = −0.31, *p* = 0.015, and 95%CI: −0.56 −0.08). Also, the visual motor imagery was positively and significantly related to coordination (β = 0.30, *p* = 0.018, and 95%CI: 0.05 0.53). On the other hand, no significant paths were confirmed for the relations kinaesthetic motor imagery with reaction time (β = 0.10, *p* = 0.460, and 95%CI: −0.15 0.33) and coordination (β = −0.02, *p* = 0.877, and 95%CI: −0.26 0.23). Furthermore, there was no relation between coordination and the trainer’s assessment tactics (β = −0.18, *p* = 0.252, and 95%CI: −0.48 0.13) and technique (β = −0.17, *p* = 0.265, and 95%CI: −0.48 0.13). Also, no significant paths were confirmed for the relation between reaction time and the trainer’s assessment tactics (β = −0.26, *p* = 0.096, and 95%CI: −0.55 0.05) and technique (β = −0.27, *p* = 0.094, and 95%CI: −0.56 0.04). There was a significant positive correlation between residuals of KMI and VMI (r = 0.39, *p* = 0.001, and 95%CI: 0.17 0.57). In addition, negative significant correlation was confirmed for relation between residuals of reaction time and coordination (r = −0.61, *p* < 0.001, and 95%CI: −0.74 −0.43). Also, there was a significant positive correlation between residuals of the trainer’s assessment tactics and technique (r = 0.83, *p* < 0.001, and 95%CI: 0.74 0.89). Detailed findings are shown in Figure 2.

## 4. Discussion

The study confirmed the significant relationship between the VMI scale and two fundamental motor skills, simple visual reaction time and eye–hand coordination, in young soccer players. These results can be interpreted in the context of data from a group of adult athletes. The primary role of VMI over KMI has been recognised in other target-oriented sporting disciplines, like darts [14] and volleyball [15]. It is possible that in a group of soccer players, the ability to see the action from the perspective of a third person is more strongly associated with motor performance than with the kinesthetic experience. During the game, most of the time, players must anticipate and react to the movement of their opponents. Therefore, it would be valuable to test whether a different relationship between KMI, VMI, and fundamental motor skills would be observed in sports disciplines where kinesthetic feeling plays an important role, e.g., wrestling.

Fitts and Peterson [47] claimed that visual control is important during learning a new perceptual-motor-task, but when the skill is integrated, the kinesthetic modality becomes more relevant. In another study, Fleishman and Rich [48] correlated the 10 runs of two-hand coordination (THC) test with spatial-visual (aerial orientation) and kinesthetic sensitivity (estimating the weight) tasks. The results confirmed Fitts et al.’s assumptions because kinesthetic sensitivity was significantly correlated later with perceptual-motor learning (after seven runs) than spatial-visual skills (first three runs). It is possible that the same mechanism was observed in the current study. The assessment of reaction time and coordination was a new perceptual-motor-task for the participants, promoting people with higher visual and spatial abilities. This trend could be reversed if motor skills were measured over a longer time, e.g., after the training. In the previous study [49], after MI training, the KMI rather than VMI vividness was a predictor of sequential finger movements performance. A similar effect was also observed in adult tennis players with high KMI scores who have improved their performance as a result of MI training [50].

On the other hand, some findings [51] suggest that VMI correlates with performance on tasks where execution is relevant (like drawing) while KMI where speed and coordination are essential. A possible explanation for this inconsistency could be that the current study measured simple motor skills requiring mostly automatic responses. Perhaps when more advanced motor abilities—involving, for example, identification or differentiation of stimuli—would be measured, the role of KMI could be revealed.

An alternative explanation for the lack of relevance of KMI in the model structure is the difference in developing these two perspectives in children and young adolescents. In a validation study of MIQ-C questionnaire, Martini et al. [31] have observed that external visual imagery was reported as the easiest perspective to imagine while kinesthetic imagery was reported as the most difficult. Other researchers showed that young athletes used imagery to focus on a goal [30] rather than solve problems in this way [52]. The use of kinesthetic images may be less common in the studied age range. The role of development processes in this case is also supported by the fact that players of this age in their sports training do not yet apply mental practice, which is often based on visualization [3,4]. Moreover, a study involving neurophysiological methods, such as magnetoencephalography, showed some linear changes in the organization of movement-related brain activity in school-aged children at a different age [53]. The study showed that the magnitude of mu (8–12 Hz) and beta (15–30 Hz) bands are linear increases with age. At the same time, both oscillations were associated with brain activity during kinesthetic and visual-motor imagery [13]. It is possible that the movement-related imagery can also undergo developmental changes caused by changes in the organization of the central nervous system. So far, there are no similar longitudinal studies showing changes in KMI and VMI in children and adolescents. The application value of the results is that the trainers can look for reserves in the KMI area. An early start to KMI training may give a better chance of sports success in the future.

Additionally, developmental changes related to motor imagery can intertwine with the training systems of young soccer players. More precisely, incorporating visualization training in young soccer players leads to improved task performance [54]. However, depending on the motor imagery preference by individuals, it can have different effects [55]. In this context, Bahmani et al. [55] showed that higher levels of external-visual imagery dominance lead to greater motor learning associated with an external focus among children. However, they also reported that higher kinesthetic imagery dominance levels reduced motor learning in children associated with external focus. Consequently, it can be assumed that the implementation of visualization training based on visual imagery in young soccer players may contribute to better performance in motor tasks in individuals who prefer this motor imagery type.

The lack of relation between motor skills and the coach’s assessment was also observed in other studies with the young athletes (for example: [37]). The trainers’ evaluations may also be influenced by other factors, such as motivation, mental predisposition, etc. A strong relationship between the tactics and technique assessments (0,83) may suggest that the coaches rate the players without distinguishing between these two aspects. If a player is well-rated, all aspects of his efficiency are also valued high. Perhaps an objective performance test would be a better measure of the players’ tactics and technique than the coach’s assessment.

Some limitations of the study should also be mentioned. First, the age range of participants was quite wide. Because of the high dynamics of developmental change in early adolescence, it would be appropriate to conduct analyses in more narrow age groups. Considering the difference between closed and open sports in VMI [56], it is important to be cautious about generalizing the present results conducted on the soccer player group to other sports groups. The results may also have been affected by the small group size, making it difficult to obtain conclusive effects of the statistical analyses.

## 5. Conclusions

The present study results provide new evidence that visual MI rather than kinesthetic MI is associated with simple visual reaction time and eye–hand coordination in the group of young soccer players. A possible explanation for this result may be the unique role of the third-person perspective in soccer or the developmental changes in brain areas responsible for movement-related activity. The results of other studies suggest that during the performance of a new activity, the third-person perspective in imagery is more relevant than during the further stages of the same motor actions. However, all these explanations require empirical verification, mainly because studies on mental visualization in young athletes are still rare.

Results obtained in the current study are the valuable starting point for future research on the relationship between motor imagery abilities and motor performance in various age groups and disciplines. In particular, future research should focus on investigating the role of VMI and KMI in individual and team sports and disciplines that require more visual analysis versus disciplines where the dominant role plays on sensation of touch. A better understanding of the relationships among mental and motor activity can bring significant progress in diagnosing sports predispositions and the training process. In this context, the importance of VMI and KMI training in improving athletes’ abilities [10,50,57] should be noted. Consequently, understanding the relationship between KMI, VMI, and motor performance for different sport groups can contribute to preparing specific and detailed imagery training protocols for each particular sport, especially for soccer players.

## Data Availability

The data presented in this study are available on request from the corresponding author. The data are not publicly available due to the risk of identification of study participants.

## Appendix A

**Table A1:** Player’s assessment survey.

The Trainer’s Assessment Tactics and Technique										
1 Overall Assessment Of The Player’s Physical Condition	1	2	3	4	5	6	7	8	9	10
2 Overall Assessment Of The Player’s Technical Skills	1	2	3	4	5	6	7	8	9	10
3 Overall Assessment Of The Player’s Tactical Skills	1	2	3	4	5	6	7	8	9	10
4 Global Assessment Of The Player	1	2	3	4	5	6	7	8	9	10

Please indicate your answer by marking the correct number on the scale next to each question.

## References

[1] Jeannerod M. (2001). Neural simulation of action: A unifying mechanism for motor cognition. Neuroimage.

[2] Hall C.R., Rodgers W.M., Barr K.A. (1990). The use of imagery by athletes in selected sports. Sport Psychol..

[3] Frank C., Land W.M., Popp C., Schack T. (2014). Mental Representation and Mental Practice: Experimental Investigation on the Functional Links between Motor Memory and Motor Imagery. PLoS ONE.

[4] Frank C., Land W.M., Schack T. (2016). Perceptual-Cognitive Changes during Motor Learning: The Influence of Mental and Physical Practice on Mental Representation, Gaze Behavior, and Performance of a Complex Action. Front. Psychol..

[5] Suinn R.M. (1997). Mental Practice in Sport Psychology: Where Have We Been, Where Do We Go?. Clin. Psychol..

[6] Jones L., Stuth G. (1997). The Uses of Mental Imagery in Athletics an Overview. Appl. Prev. Psychol..

[7] Ridderinkhof K.R., Brass M. (2015). How kinesthetic motor imagery works: A predictive-processing theory of visualization in sports and motor expertise. J. Physiol. Paris.

[8] Lotze M., Halsband U. (2006). Motor imagery. J. Physiol. Paris.

[9] Ungerleider S., Golding J.M. (1991). Mental practice among Olympic athletes. Percept. Mot. Ski..

[10] Lebon F., Collet C., Guillot A. (2010). Benefits of motor imagery training on muscle strength. J. Strength Cond. Res..

[11] Grosprêtre S., Jacquet T., Lebon F., Papaxanthis C., Martin A. (2018). Neural mechanisms of strength increase after one-week motor imagery training. EJSS.

[12] Yágüez L., Nagel D., Hoffman H., Canavan A.G.M., Wist E., Hömberg V. (1998). A mental route to motor learning: Improving trajectorial kinematics through imagery training. Behav. Brain Res..

[13] Neuper C., Scherer R., Reiner M., Pfurtscheller G. (2005). Imagery of motor actions: Differential effects of kinesthetic and visual-motor mode of imagery in single-trial EEG. Cogn. Brain Res..

[14] Cumming J., Nordin S.M., Horton R., Reynolds S. (2006). Examining the direction of imagery and self-talk on dart-throwing performance and self efficacy. Sport Psychol..

[15] Filgueiras A. (2017). Imagery for the Improvement of Serving in Beach Volleyball: A Single Case Study. Rev. Bras. Psicol. Esporte.

[16] Féry Y.A., Morizot P. (2000). Kinesthetic and visual image in modeling closed motor skills: The example of the tennis serve. Percept. Mot. Ski..

[17] Hétu S., Grégoire M., Saimpont A., Coll M.P., Eugène F., Michon P.E., Jackson P.L. (2013). The neural network of motor imagery: An ALE meta-analysis. Neurosci. Biobehav. Rev..

[18] Filgueiras A., Conde E.F.Q., Hall C.R. (2017). The neural basis of kinesthetic and visual imagery in sports: An ALE meta-analysis. Brain Imaging Behav..

[19] Guillot A., Collet C., Nguyen V.A., Malouin F., Richards C., Doyon J. (2008). Brain activity during visual versus kinesthetic imagery: An fMRI study. Hum. Brain Mapp..

[20] Funk M., Brugger P., Wilkening F. (2005). Motor processes in children’s imagery: The case of mental rotation of hands. Dev. Sci..

[21] Krüger M., Krist H. (2009). Imagery and motor processes—When are they connected? The mental rotation of body parts in development. Cogn. Dev..

[22] Molina M., Tijus C., Jouen F. (2008). The Emergence of Motor Imagery in Children. J. Exp. Child Psychol..

[23] Smits-Engelsman B., Wilson P. (2012). Age-related changes in motor imagery from early childhood to adulthood: Probing the internal representation of speed-accuracy trade-offs. Hum. Mov. Sci..

[24] Spruijt S., van der Kamp J., Steenbergen B. (2015). Current insights in the development of children’s motor imagery ability. Front. Psychol..

[25] Butson M., Hyde C., Steenbergen B., Williams J. (2014). Assessing motor imagery using the hand rotation task: Does performance change across childhood?. Hum. Mov. Sci..

[26] Caeyenberghs K., Tsoupas J., Wilson P.H., Smits-Engelsman B.C. (2009). Motor imagery development in primary school children. Dev. Neuropsychol..

[27] Hoyek N., Champely S., Collet C., Fargier P., Guillot A. (2009). Age and gender-related differences in the temporal congruence between motor imagery and motor performance. Learn. Individ. Differ..

[28] Skoura X., Vinter A., Papaxanthis C. (2009). Mentally simulated motor actions in children. Dev. Neuropsychol..

[29] Choudhury S., Charman T., Bird V., Blakemore S.-J. (2007). Development of action representation during adolescence. Neuropsychologia.

[30] Gregg M., Strachan L. (2015). Examining Developmental Differences in Imagery Use with Youth Soccer Players. J. Imag. Res. Sport Phys. Act..

[31] Martini R., Carter M.J., Yoxon E., Cumming J., Ste-Marie D.M. (2016). Development and validation of the Movement Imagery Questionnaire for Children (MIQ-C). Psychol. Sport Exerc..

[32] Simonsmeier B.A., Frank C., Gubelmann H., Schneider M. (2018). The effects of motor imagery training on performance and mental representation of 7-to 15-year-old gymnasts of different levels of expertise. Sport Exerc. Perform. Psychol..

[33] Li-Wei Z., Qi-Wei M., Orlick T., Zitzelsberger L. (1992). The effect of mental-imagery training on performance enhancement with 7–10-year-old children. Sport Psychol..

[34] Smith D., Wright C., Allsopp A., Westhead H. (2007). It’s all in the mind: PETTLEP-based imagery and sports performance. J. Appl. Sport Psychol..

[35] Mori S., Ohtani Y., Imanaka K. (2002). Reaction times and anticipatory skills of karate athletes. Hum. Mov. Sci..

[36] Lopes V., Rodrigues L., Maia J.A., Malina R.M. (2011). Motor coordination as predictor of physical activity in childhood. Scand. J. Med. Sci. Sports.

[37] Vandorpe B., Vandendriessche J.B., Vaeyens R., Pion J., Lefevre J., Philippaerts R.M., Lenoir M. (2012). The value of a non-sport-specific motor test battery in predicting performance in young female gymnasts. J. Sports Sci..

[38] Nuri L., Shadmehr A., Ghotbi N., Attarbashi Moghadam B. (2013). Reaction time and anticipatory skill of athletes in open and closed skill-dominated sport. EJSS.

[39] Zazzo R. (1979). Manuel Pour L’examen Psychologique De L’enfant. Tome 1.

[40] Bogdanowicz M. (1989). Diagnosis and Treatment of Children with Reading and Writing Difficulties in Poland. Int. J. Disabil. Dev. Educ..

[41] Gregg M., Hall C., Butler A. (2010). The MIQ-RS: A Suitable Option for Examining Movement Imagery Ability. eCAM.

[42] Naglak Z. (1999). Metodyka Trenowania Sportowca.

[43] Nevitt J., Hancock G.R. (2004). Evaluating small sample approaches for model test statistics in structural equation modeling. Multivar. Behav. Res..

[44] Kline R.B., Kline R.B. (2011). Principles and Practice of Structural Equation Modeling.

[45] Byrne B.M., Byrne B.M. (2010). Structural Equation Modeling with AMOS: Basic Concepts, Applications, and Programming.

[46] Kwiatkowski M., Borek D., Żukowski N. (2006). Norm klasyfikacyjne czasów reakcji dla grupy sportowców. Sport Wyczyn..

[47] Fitts P.M., Peterson J.R. (1964). Information capacity of discrete motor responses. J. Exp. Psychol..

[48] Fleishman E.A., Rich S. (1963). Role of kinesthetic and spatial-visual abilities in perceptual-motor learning. J. Exp. Psychol..

[49] Lebon F., Horn U., Domin M., Lotze M. (2018). Motor imagery training: Kinesthetic imagery strategy and inferior parietal f MRI activation. Hum. Brain Mapp..

[50] Nicolas R., Laurent D., Toussaint L., Blandin Y. (2007). Effects of motor imagery training on service return accuracy in tennis: The role of imagery ability. Int. J. Sport Exerc. Psychol..

[51] Féry Y.A. (2003). Differentiating visual and kinesthetic imagery in mental practice. Can. J. Exp. Psychol. Rev. Can. Psychol. Exp..

[52] Veraksa A., Gorovaya A. (2012). Imagery training efficacy among novice soccer players. Procedia-Soc. Behav. Sci..

[53] Johnson B., Jobst C., Al-Loos R., He W., Cheyne D. (2019). Developmental Changes in Movement Related Brain Activity in Early Childhood. BioRxiv.

[54] Slimani M., Bragazzi N.L., Tod D., Dellal A., Hue O., Cheour F., Taylor L., Chamari K. (2016). Do cognitive training strategies improve motor and positive psychological skills development in soccer players? Insights from a systematic review. J. Sports Sci..

[55] Bahmani M., Babak M., Land W.M., Howard J.T., Diekfuss J.A., Abdollahipour R. (2021). Children’s motor imagery modality dominance modulates the role of attentional focus in motor skill learning. Hum. Mov. Sci..

[56] Yu Q.H., Fu A.S., Kho A., Li J., Sun X.H., Chan C.C. (2016). Imagery perspective among young athletes: Differentiation between external and internal visual imagery. J. Sport Health Sci..

[57] Callow N., Jiang D., Roberts R., Edwards M.G. (2017). Kinesthetic imagery provides additive benefits to internal visual imagery on slalom task performance. J. Sport Exerc. Psychol..

